# HER2-targeting CAR-T cells show highly efficient anti-tumor activity against glioblastoma both in vitro and in vivo

**DOI:** 10.1038/s41435-024-00275-6

**Published:** 2024-05-03

**Authors:** Xueying Li, Lifen Zhao, Wenzhe Li, Peng Gao, Nianzhu Zhang

**Affiliations:** 1 Clinical Laboratory, The Second Hospital of Dalian Medical University, Dalian, Liaoning Province 116023 China; 2 Research Institute for Microbial Diseases and World Premier International Immunology Frontier Research Center, Osaka University, 3-1 Yamadaoka Suita, Osaka, 565-0871 Japan; 3 Guangdong Provincial Key Laboratory of Infectious Diseases and Molecular Immunopathology, Shantou University Medical College, Shantou, Guangdong Province 515041 China

**Keywords:** Immunosuppression, Immunoediting

## Abstract

Glioblastoma (GBM) is the most common and aggressive malignant primary brain tumor in adults. Current treatment options for GBM include surgical resection, radiation, and chemotherapy, which predominantly slow cancer growth and reduce symptoms, resulting in a 5-year survival rate of no more than 10%. Chimeric antigen receptor (CAR) T-cell therapy is a new class of cellular immunotherapy that has made great progress in treating malignant tumors. Human epidermal growth factor receptor 2 (HER2) is overexpressed in GBM and may provide a potential therapeutic target for GBM treatment. In this study, we constructed third-generation CAR-T cells targeting the HER2 antigen in GBM. HER2-CAR-T cells showed effective anti-tumor activity both in vitro and in vivo. Furthermore, HER2-specific CAR-T cells exhibited strong cytotoxicity and cytokine-secreting abilities against GBM cells in vitro. Anti-HER2 CAR-T cells also exhibited increased cytotoxicity with increasing effector-to-target ratios. Anti-HER2 CAR-T cells delivered via peritumoral injection successfully stunted tumor progression in vivo. Moreover, peritumoral intravenous administration of anti-HER2 CAR-T cells resulted in therapeutic improvement against GBM cells compared with intravenous administration. In conclusion, our study shows that HER2 CAR-T cells represent an emerging immunotherapy for treating GBM.

## Introduction

Glioblastoma multiforme (GBM) is the most common type of malignant (cancerous) brain tumor. Glioblastoma can occur at any age but tends to occur more often in older adults and men. All glioblastomas are grade IV brain tumors containing the most abnormal and aggressive cells. Glioblastoma symptoms include headaches that continue to worsen, nausea, vomiting, blurred or double vision, and seizures. The disease’s five-year survival rate is approximately 10%, with a median of 15- to 18-month survival after diagnosis. Currently, standard clinical treatments include maximal safe surgical resection, radiation, and chemotherapy and are mostly limited by low therapeutic efficiency correlated with poor prognosis [1]. Treatment is difficult because tumor cells are resistant to conventional therapies [2]. Furthermore, many drugs cannot cross the blood-brain barrier to act on the tumor [3]. In addition, Solid tumors are heterotypic aggregates of many cell types. Consequently, all glioblastomas recur, grow quickly, and invade and destroy healthy tissues. Thus, novel treatments for GBM are urgently needed.

Chimeric antigen receptor (CAR) T-cell therapy is a revolutionary new pillar of cancer treatment [4]. CAR-T cells effectively kill tumor cells by specifically recognizing and binding to antigens on their membranes [5]. CARs are engineered synthetic receptors that redirect lymphocytes, most commonly T cells, to recognize and eliminate cells expressing a specific target antigen independent of the major histocompatibility complex [4, 6]. CAR-T immunotherapy is commonly used to treat hematological malignancies such as acute lymphoblastic leukemia (ALL), chronic lymphocytic leukemia, lymphoma, and multiple myeloma [7]. Currently, available CAR-T therapies target the cluster of differentiation (CD)19 antigen [8]. An ideal target should have high levels of surface expression, tissue specificity, and stability to ensure the effectiveness and tolerability of CAR-T cells [9]. The most frequently used target is CD19 on B cells, predominantly used for treating lymphoma and ALL, leading to the approval of CAR-T cell therapies for clinical applications by the US Food and Drug Administration. Another US Food and Drug Administration-approved CAR-T cell-based therapy that targets B cell tumor antigens is B cell maturation antigen (BCMA)-specific CAR-T cells, which are approved for treating multiple myeloma. Other targets included CD20, CD22, CD23, receptor tyrosine kinase-like orphan receptor 1, CD4, CD30, CD33, glucose-regulated protein 78, signaling lymphocytic activation molecule family, and CD138. CAR-T cell therapy has achieved success in the context of hematological malignancies; however, an increasing number of trials are being conducted in patients with solid tumors. There are fundamental barriers to CAR-T therapy in solid tumors compared with hematological malignancies. One barrier to the effectiveness of cell therapy against solid tumors is antigen heterogeneity, which impairs the detection of cancer cells by T cells and reduces the impact of CAR-T therapy [10]. The treatment of solid tumors is also limited by the inadequate transport and infiltration capability of CAR-T cells due to the physical tumor barriers (such as the tumor stroma) that restrict the penetration and mobility of CAR-T cells [10]. In addition to physical barriers, T cells must confront highly immunosuppressive tumor microenvironments with cellular, molecular, and metabolic profiles that ultimately lead to T cell exhaustion and dysfunction [11]. According to the above analysis, solid tumors may exhibit cell-intrinsic resistance mechanisms against CAR-T cell cytotoxicity [12].

GBM remains incurable despite the aggressive implementation of multimodal treatment after surgical debulking. Almost all patients with GBM relapse within a narrow margin around the initially resected lesion because of the post-surgery residual glioma stem cells [13]. Owing to the specific localization of tumors in the brain and their inherent resistance to conventional therapy, GBMs present unique treatment challenges. Therefore, there is an urgent need to develop new strategies and delivery modalities for treating GBMs. Previous studies have acknowledged the potential of CAR-T cells for the treatment of glioblastoma. CAR-T cell treatments targeting GBM have been the subject of many clinical studies. Currently, many targets, such as epidermal growth factor receptor variant III, interleukin (IL)–13Rα2, B7 homolog 3 protein (also known as CD276), CD70, ganglioside GD2, matrix metalloproteinase-2, and natural killer group 2, member D, have been used in preclinical and clinical studies of CAR-T therapy conducted on GBM [14]. The epidermal growth factor receptor family member epidermal growth factor receptor 2 (HER2), also known as ErbB2, is overexpressed in approximately 80% of GBM [15]. High expression of HER2 has been associated with GBM development and progression [16]. The ability of HER2-specific CAR-T cells to eliminate both differentiated GBM cells and GBM-initiating cells makes them attractive target tumor antigens [17]. The traditional route of CAR-T treatment for GBM involves intravenous delivery. Several medications, including CAR-T cells, are prevented from entering the brain because of the unique structure of the blood-brain barrier. When targeting HER2, the main focus is on the potential side effects caused by the expression of HER2 in various normal tissues, especially in important organs [18]. Therefore, a locoregional treatment strategy with improved efficacy and prognosis for HER2-CAR-T cell-specific tumoricidal immunity needs to be explored.

In this study, HER2-targeted CAR-T (HER2-CAR-T) cells were engineered, and their anti-tumor effects on GBM were evaluated both in vitro and in vivo. HER2-CAR-T cells exhibited strong cytotoxicity and cytokine secretion efficiency against GBM cells in vitro. Furthermore, HER2-CAR-T cells delivered by peritumoral injection showed greater therapeutic improvement against GBM cells than those delivered via tail intravenous administration. Consequently, HER2-CAR-T cells showed high anti-tumor efficacy against GBM, suggesting that peritumoral injection has enormous potential as a CAR-T cell treatment strategy.

## Materials and methods

### Construction of the HER2-, mesothelin (MSLN)-, and epithelial cellular adhesion molecule (EpCAM)-targeting CAR vectors

HER2-specific, MSLN-specific, and EpCAM-specific single-chain antibody fragment variants (scFvs) with a CD8 leading sequence, a CD8 hinge, and transmembrane sequence, as well as the intracellular signaling domain of 4-1BB, CD28, and CD3ζ in tandem, were engineered into third generation HER2-, MSLN-, and EpCAM-CAR T cells. Except for scFv, the sequences have been previously reported [19]. The full-length nucleotide sequence was synthesized by Sangon Biotech (Shanghai, China) and inserted into the CAR lentiviral expression vector [pCDH-EF1-MCS-EF1-puro] at two specific restriction enzyme sites (*EcoR1* and *Not1*).

### Establishment of HER2-targeted CAR-T cells

The pCDH-CMV-MCS-EF1-puro lentivirus system was used to generate a virus against the HER2-targeted CAR. HEK293T cells were co-transfected with a HER2-targeting CAR vector or control vector, with PLP1, PLP2, and PLP-VSVG at a ratio of 23.1:16.5:16.5:9.9, polyethyleneimine (Polysciences, Warrington, PA, USA). Six hours after transfection, the cells were re-fed with Dulbecco’s modified Eagle’s medium supplemented with 20% fetal bovine serum. After transfection, cell supernatant was collected at 48 and 72 h, centrifuged at 4000 rpm for 5 min to remove cell debris, and filtered through a 0.45 μm filter. To concentrate the virus, 1:4 PEG8000 (Sigma, Merk, Shanghai, China) was added and mixed four times every 30 min. After that, the virus was placed overnight at 4 °C and centrifuged at 4000 × *g* for 30 min. The supernatant was removed, and lentivirus particles were resuspended in phosphate-buffered saline (PBS) and stored at −80 °C.

Human peripheral blood mononuclear cells were isolated through density gradient centrifugation with a Ficoll kit (GE, Shanghai, China) and subsequently activated with anti-human CD3 (100 ng/mL; T&L Biotechnology) and anti-human CD28 (100 ng/mL; T&L Biotechnology). Recombinant human IL-2 (30 ng/mL; Novoprotein) and 1% penicillin–streptomycin (Gibco, Life Technologies, Shanghai, China) were added to X-VIVO 15 medium (Lonza, USA) for T-cell proliferation. After activation for 24 h, T cells were transduced with HER2-targeted CAR lentiviral particles, selected using puromycin, and collected for subsequent in vivo and in vitro experiments after 12-14 days. Non-transduced T cells were used as a control group.

### Cell lines and culture conditions

HOS, U118MG, U251, U87MG, HepG2, MKN-45, and SK-OV-3 cells were obtained from the American Type Culture Collection (USA). HOS and SK-OV-3 cells were maintained in Roswell Park Memorial Institute 1640 medium (Gibco), whereas 293 T, U118MG, U251, U87MG, HepG2, and MKN-45 cells were maintained in Dulbecco’s modified Eagle’s medium (Gibco). All cells were cultured in a medium supplemented with 10% fetal bovine serum (BI, China), penicillin (60 μg/mL), and streptomycin (100 μg/mL) (Sangon, China) and maintained in an incubator with 5% CO_2_ at 37 °C.

### Immunohistochemistry

Human glioblastoma tissues were obtained from the Second Hospital of Dalian Medical University. Surgical glioblastoma tissues were fixed in 4% paraformaldehyde and embedded in paraffin. Then, 4 μm thickness sections were deparaffinized in xylene and rehydrated with 100, 90, 80, and 70% ethanol to PBS. The HER2 antibody (Cell Signaling Technology, USA) was used for immunostaining at room temperature for 2 h. After the slides were incubated with the HRP-labeled secondary antibodies at room temperature for 1 h, 3,3′-diaminobenzidine was added for coloration. The staining intensity was analyzed using integrated optical density using the Image-Pro R Plus software (version 6.0; Media Cybernetics, USA).

### Flow cytometry and antibodies

Cells were incubated with antiCD16/CD32 (2.4G2) monoclonal antibody to block Fcγ receptors. Recombinant anti-HER2 FITC-conjugated antibody and recombinant anti-EpCAM FITC-conjugated antibody (Sino Biological Inc., Beijing, China) were used to detect HER2 and EpCAM protein expression, respectively. A recombinant anti-MSLN FITC antibody (Abcam, Cambridge, UK) was used to detect MSLN expression. Cells were stained with antibodies against HER2, EpCAM, or MSLN for 1 h on ice. CAR expression in CAR-T cells was detected using biotinylated human HER2/MSLN/EpCAM (Acro, Beijing, China), followed by staining with allophycocyanin streptavidin (BioLegend, CA, USA).

Anti-mouse CD3 (17A2, BioLegend, USA) was used to detect the presence of CD3^+^ T cells in the mouse peripheral blood. A FACSCalibur (Becton Dickinson, USA) was used to perform flow cytometry according to prior guidelines [20], and FlowJo software (Tree Star) was used to analyze the data.

### Cytotoxicity assays

Tumor cells were regarded as target cells (T) and were suspended at a density of 2 × 10^5^ cells/mL. Then, 0.1 mL of the cell suspension was transferred to a 96-well e-plate (ACEA Biosciences, Menlo Park, CA, USA) and cultured for 20 h. Then, HER2-CAR-T and untransfected T cells (NC-T) were regarded as effector cells (E) and added to each well separately at different E:T ratios (5:1 or 2.5:1). The co-cultures were further cultured for the indicated periods. RTCA software (xCELLigence RTCASP, ACEA, Los Angeles, CA, USA) was used to measure the viability of the target cells in real time.

### Measurement of cytokine secretion

HER2-CAR-T and NC-T cells were co-cultured with tumor cells for 24 h in a 96-well plate without added cytokines. Enzyme-linked immunosorbent assay (ELISA) kits (eBioscience, Grand Island, NY, USA) for specific cytokines (interferon-gamma (IFN-γ), tumor necrosis factor-alpha (TNF-α), granulocyte macrophage colony-stimulating factor (GM-CSF), IL-6, and IL-8) were used to detect the level of cytokine production in the supernatant and assess the cell-killing efficacy.

### Tumor models and treatment

First, 6- to 8-week-old NODPrkdcem26IL2rgem26/Nju (NCG) mice were obtained from the Nanjing Biomedical Research Institute of Nanjing University and Nanjing Galaxy Biopharma (Nanjing, China). Mice were maintained at 24 ± 1 °C with free water and food intake and illuminated for 12 h (08:00 to 20:00) in the specific pathogen-free laboratory animal facility of Dalian Medical University (Dalian, China).

For cell-derived xenograft (CDX) mouse models, 1 × 10^6^ U118MG tumor cells in 100 μL of PBS were injected subcutaneously (*s.c*.) into the axilla of NCG mice. The tumor size was measured every four days. The mice were divided into three groups, with six mice per group until the tumor volume reached 50–100 mm^3^. Tumor volumes were calculated using the following formula: tumor volume = (length) × (width)^2^ × 0.5, where length represents the longer dimension, and tumor weights were recorded. The mice were monitored according to the Institutional Animal Care and Use Committee animal facility rules and regulations. Situations where the experiment needed to be paused immediately were listed as previously reported [21].

For in vivo tumor killing, HER2-CAR-Ts were administered via two methods: peritumoral (*p.v*.) and intravenous injection (*i.v*.). Each U118MG-CDX mouse model was injected with 5 × 10^6^ HER2-CAR-T cells in 200 μL of PBS. Non-injected mice (non-transduced T cells, NC-T) were used as controls.

### Statistical analysis

Data are presented as mean ± standard deviation (SD) from at least three experiments. Statistical analysis was performed via unpaired *t* test or ANOVA as indicated and reported as mean ± SD. Analyses were done using GraphPad Prism software version 9 and SPSS Statistics 26. *p* < 0.05 was considered to indicate statistical significance. *p < 0.05, **p < 0.01, ***p < 0.001.

## Results

### Expression of HER2 in glioblastoma samples and tumor cell lines

**Fig. 1:**
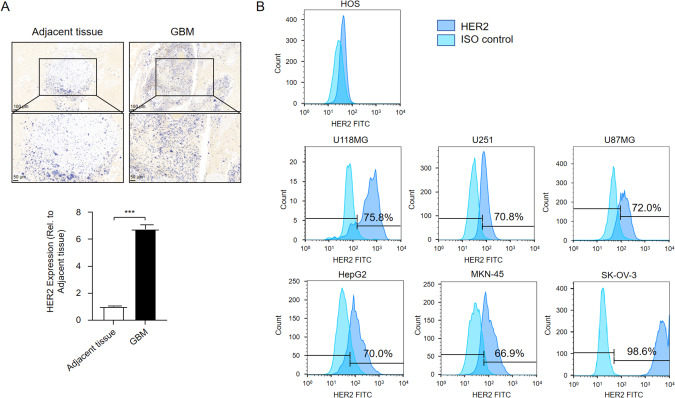
Expression of HER2 in GBM samples and tumor cell lines. **A** Representative images of immunohistochemical analysis showing HER2 levels in tumor and adjacent tissue from patients with GBM. After washing, the secondary antibody was incubated and visualized with 3,3′-diaminobenzidine. Light yellow indicates low expression, and deep yellow indicates high expression. GBM, glioblastoma. Scale bar = 50 and 100 μm. **B** Surface expression of HER2 on tumor cell lines (U118MG, U251, U87MG, HepG2, MKN-45, SK-OV-3, and HOS). HER2 expression on the cell surface was verified using flow cytometry. Numbers indicate the percentage of HER2^+^ cells, and 10,000 events were acquired for each analysis.

To understand the relationship between HER2 expression and glioblastoma, immunohistochemical analysis of surgical glioma sections was performed with HER2 antibody. The representative immunohistochemical results are shown in Fig. 1A. HER2 was highly expressed in glioma cells. To further determine whether HER2 could be a therapeutic target for tumor therapy, HER2-positive cancer cells were selected using flow cytometry. HER2 was highly expressed in the glioblastoma cell lines U118MG, U251, and U87MG (Fig. 1B). In addition, HER2 expression in other types of tumor cells was detected in subsequent studies. Hepatocellular carcinoma (HepG2), human gastric carcinoma (MKN-45), and human ovarian adenocarcinoma (SK-OV-3) cells showed high HER2 protein expression (Fig. 1B). Human osteosarcoma cells (HOS) cells were used as negative controls (Fig. 1B). Collectively, these data suggest a widespread expression of HER2 in different tumor cell types.

### Construction of third-generation CAR-T cells targeting HER2

**Fig. 2:**
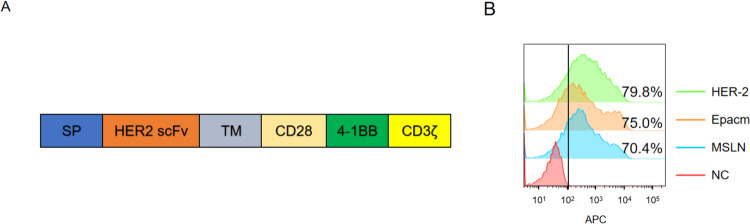
Construction of 3rd-generation CAR-T cells targeting HER2. **A** Schematic diagram of the HER2 CAR-T transgene. B. HER2, EpCAM, and MSLN expression was measured using flow cytometry. CAR-T cells were incubated with biotinylated anti-HER2, anti-MSLN, and anti-EpCAM, followed by incubation with APC streptavidin (1:1000). Data (**B**) is shown as mean ± SD of triplicates and is representative of three independent experiments.

Given the importance of HER2 in glioblastomas and other tumors, we generated a lentiviral CAR expression plasmid targeting human HER2 using genetic engineering. HER2-CARs include three main parts: an extracellular antigen recognition domain of the scFv derived from an anti-HER2 antibody, a CD8 transmembrane domain, and an intracellular T cell activation domain of CD3ζ. Two other vital targets, EpCAM and MSLN, were constructed similarly for further analysis. Costimulatory domains included both CD28 and 4-1BB to construct CD3ζ-CD28-41BB (Fig. 2A). The lentivirus was produced by co-transfecting 293 T cells with the HER2 CAR plasmid, PLP1, PLP2, and PLP-VSVG. HER2, EpCAM, and MSLN CAR-T cells were prepared through lentiviral infection of peripheral blood mononuclear cells. The infection efficiency was evaluated using flow cytometry using anti-HER2, anti-EpCAM, and anti-MSLN antibodies (Fig. 2B).

### Cytotoxicity of HER2 CAR-T cells in glioblastoma

**Fig. 3:**
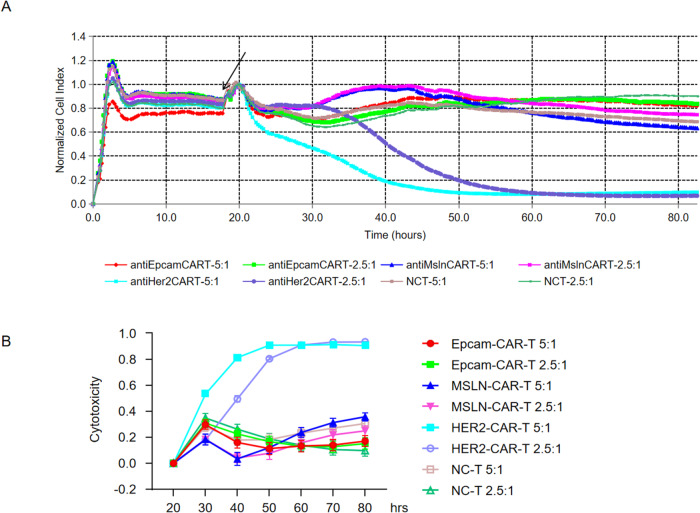
Functional study of HER2 CAR-T cells in vitro. **A** Cytotoxicity of CAR-T cells against solid tumor cell lines was analyzed using the RTCA assay. HER2-CAR-T, EpCAM-CAR-T, and MSLN-CAR-T cells were all used as effector cells. Cytotoxicity of HER2-, EpCAM-, and MSLN-CAR-T and NCT cells against U118MG cells at an E: T of 5:1 and 2.5:1 for 80 h was analyzed. Black arrow represents the time when effector cells were added. **B** Comparison of killing rates under different effector cells and E:T ratios on U118MG cells. Data are shown as mean ± SD of triplicates and represent three independent experiments.

To evaluate the cytotoxicity and specificity based on the high expression of HER2 but not other targets, CAR-T cells with different targets were co-cultured with U118MG cells. Anti-HER2 CAR-T, anti-EpCAM CAR-T, and anti-MSLN CAR-T cells were cultured for 10 days and then independently co-cultured with U118MG cells at an E: T ratio of 2.5:1 and 5:1, with the NC-T group used as the control group. Real-time cytotoxicity assays provide automated real-time data acquisition to monitor the CAR-T cell-mediated killing of cancer cells and gain deeper insights into the specificity, potency, persistence, and efficiency of CAR-T cells. As shown in Fig. 3A, compared with anti-EpCAM CAR-T and anti-MSLN CAR-T cells, HER2-CAR-T cells showed stronger cell-killing ability in U118MG cells. Moreover, the cell-killing effect at an E:T ratio of 5:1 was greater than that at an E:T ratio of 2.5:1 (Fig. 3A), indicating that HER2-CAR-T cells had a dose-dependent effect on tumor cell killing. The cell killing rate was also analyzed, as shown in Fig. 3B.

**Fig. 4:**
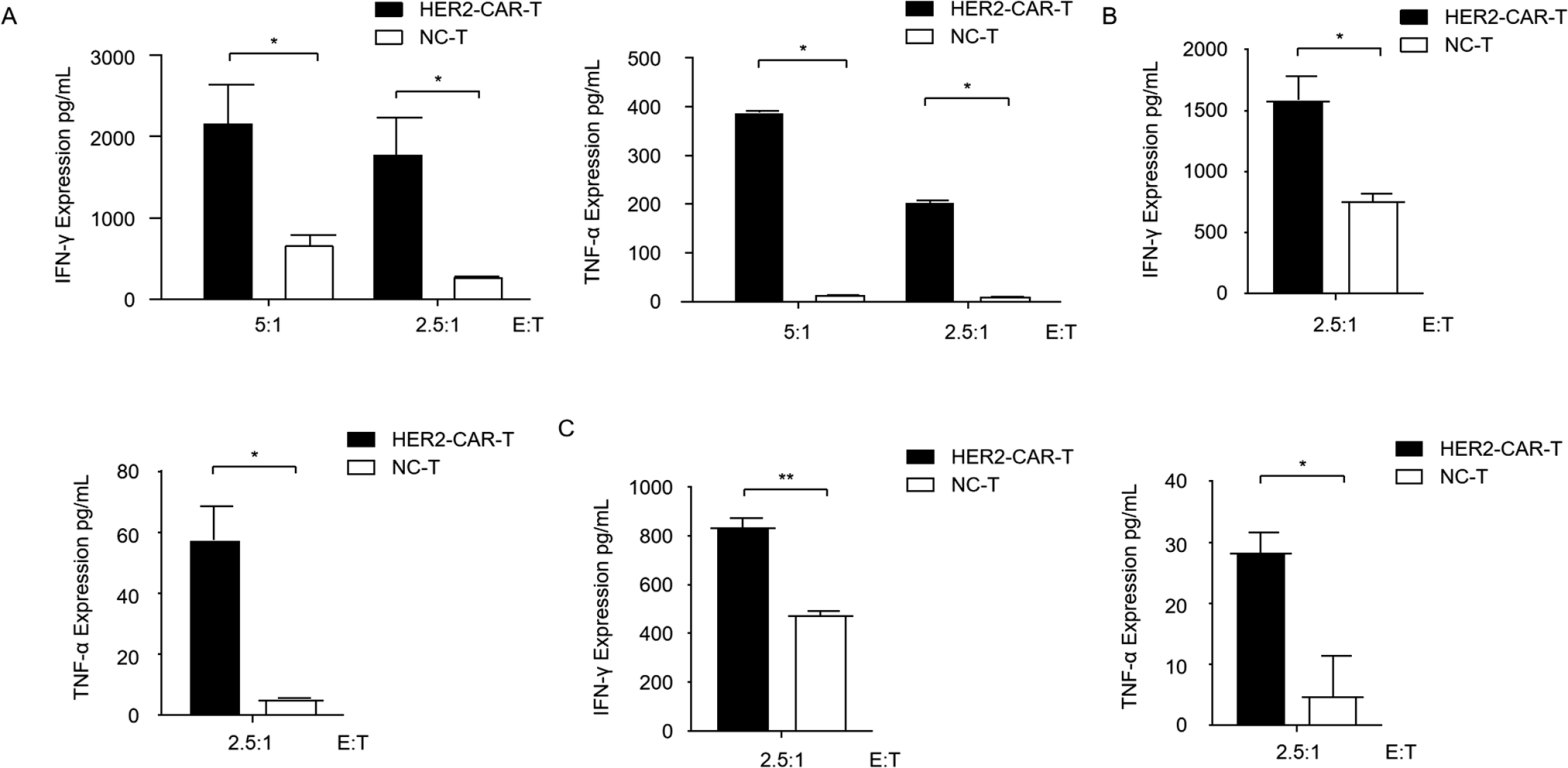
HER2 CAR-T cell-mediated cytokine release. **A** Levels of IFN-γ and TNF-α released by HER2 CAR-T cells were analyzed using ELISA after incubation with U118MG cells at an E:T of 5:1 and 2.5:1 for 20 h. Levels of IFN-γ and TNF-α released by HER2 CAR-T cells analyzed using ELISA after incubation with another two glioma cell lines U251 (**B**) and U87MG (**C**) cells at an E:T of 2.5:1 for 20 h. *p < 0.05, **p < 0.01 [Student’s *t* test (unpaired)]. Data are shown as mean ± SD of triplicates and represent three independent experiments.

It is shown that T cell-derived TNF-α and IFN-γ are required for T cell-mediated killing of established tumors [22]. Next, we compared the cytokine release mediated by HER2-CAR-T and NC-T against HER2-positive glioblastoma cell lines. IFN-γ and TNF-α released in the supernatant were all present at high levels in HER2-CAR-T-targeted U118MG, U251, and U87-MG cells compared with the corresponding NC-T targeted cells (Fig. 4A–C). These results demonstrate that the effect of HER2-CAR-T cells on tumors is target specific.

### Anti-tumor efficacy of HER2 CAR-T cells in vivo

**Fig. 5:**
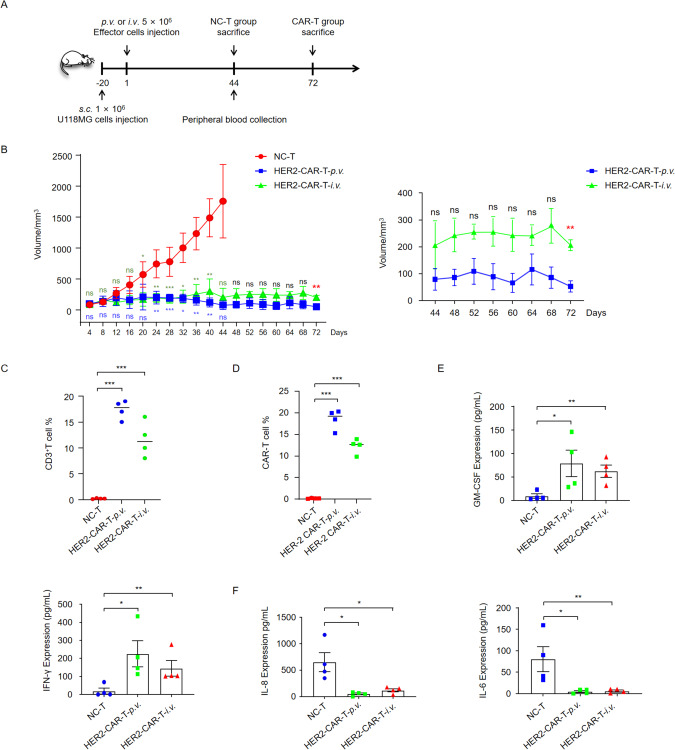
Anti-tumor efficacy of HER2-CAR-T cells against transplanted U118MG cell tumors in vivo. **A**. Experimental outline. NCG mice were divided into three groups, with six mice per group. Mice were injected *s.c*. with 1 × 10^6^ U118MG cells. Once tumors were well established on day 20, xenograft-bearing mice received 5 × 10^6^ NC-T and CAR-T cells. CAR-T cells were injected either *p.v*. or *i.v*. Mice in the NC-T group were sacrificed on day 44, while mice in the CAR-T group were sacrificed on day 72. **B**. Tumor size was measured every four days. Statistical analysis was performed with ANOVA between day 4 to day 44 and with *t* test between day 44 to day 72. The blue font indicated the analysis of NC-T versus HER2-CAR-T-*p.v*. and the dark green font indicated the analysis of NC-T versus HER2-CAR-T-*i.v*. **C**. Presence of CD3^+^ T cells in mouse peripheral blood 44 days after CAR-T cell injection. Peripheral blood mononuclear cells (PBMC) were isolated and stained with an anti-CD3 antibody. **D**. Absence of CAR-T cells in mouse tumors 44 days after injection. Tumor tissue was digested, and CD3 and HER2 double-positive cells were analyzed using polychromatic flow cytometry. Cells were probed with anti-CD3 and anti-HER2. **E**. GM-CSF and IFN-γ released by HER2 CAR-T and NC-T cells were determined using ELISA 44 days after injection. **F**. IL-8 and IL-6 released by HER2 CAR-T and NC-T cells analyzed using ELISA 44 days after injection. NS not significant, *p < 0.05, **p < 0.01, ***p < 0.001 [Student’s *t* test (unpaired) and ANOVA]. Data are shown as mean ± SD of triplicates and represent three independent experiments.

Since HER2 CAR-T cells are cytotoxic toward HER2-positive tumor cells in vitro, we speculated that HER2-CAR-T cells could also play a prominent role in tumor cell killing in vivo. We first evaluated the influence of HER2-CAR-T cells on tumor cell death in tumor-bearing mice using two injection methods (Fig. 5A). NCG mice inoculated *s.c*. with U118MG cells were randomly divided into three groups (*n* = 8): the NC-T control group (NC-T), peritumoral injection group (HER2-CAR-T-*p.v*.), and intravenous injection group (HER2-CAR-T-*i.v*.). Once the tumor size reached 50 mm^3^, 5 × 10^6^ effector cells were administered in different ways. Twenty days after effector cell injection, a significant tumor-suppressive effect was observed in both the HER2-CAR-T-*p.v*. group and HER2-CAR-T-*i.v*. group compared with the NC-T group (Fig. 5B). On day 44 after CAR-T cell implantation, NC-T mice were euthanized because of the enormous volume of tumors (Fig. 5B). The HER2-CAR-T-*p.v*. and HER2-CAR-T-*i.v*. groups were used to observe the influence of different CAR-T cell injection modes on tumor therapy. On day 44, peripheral blood was collected from each mouse, and the proportion of CD3^+^ T cells was measured. As illustrated in Fig. 5C, the proportion of CD3^+^ T cells in the peripheral blood was higher in the HER2-CAR-T-*p.v*. and HER2 CAR-T-*i.v*. groups, whereas it was depleted in the NC-T group. To further investigate the efficacy of CAR-T cells, tumor tissues were obtained surgically, and CAR-T cells were measured using flow cytometry. The proportion of CAR-T cells was maintained at high levels in the HER2-CAR-T-*p.v*. and HER2-CAR-T-*i.v*. groups compared with that in the NC-T group (Fig. 5D). The HER2-CAR-T-*p.v*. group displayed a much higher proportion of tumor-infiltrating HER2-CAR-T cells than the HER2 CAR-T-*i.v*. group, implying a better efficiency of peritumoral administration toward glioma (Fig. 5D).

To further investigate the effect of CAR-T treatment on glioma, a long-term increase in tumor volume was measured under CAR-T therapy by *p.v*. and *i.v*. Tumor volume was smaller in the HER2-CAR-T-*p.v*. group but was not statistically significant until day 72 (Fig. 5B). We subsequently measured GM-CSF and IFN-γ in the serum of HER2-CAR-T-*p.v*. and HER2-CAR-T-*i.v*. mice (Fig. 5E). Elevated GM-CSF and IFN-γ were found in the HER2-CAR-T mice, especially in HER2-CAR-T-*p.v*. mice, while IL-8 and IL-6 exhibited decreased expression (Fig. 5F). Taken together, our results suggest that CAR-T therapy by *p.v*. exerts a stronger therapeutic effect on gliomata than *i.v*. administration. Based on these results, we propose that HER2-CAR-T therapy could be considered a rational immunotherapeutic strategy, and peritumoral administration could be an innovative and locoregional approach for GBM treatment.

## Discussion

In our study, we found anti-tumor activity of HER2-CAR-T cells against HER2-positive GBM cells and other HER2-positive tumor cell types. However, high tumor heterogeneity, local physical barriers, a hostile tumor microenvironment, and antigen escape make CAR-T therapy challenging [23]. The survival of GBM cells in the harsh local environment of the brain and the elimination of multiple types of tumor antigens also pose unique difficulties that need to be solved. Tumor-associated antigens (TAA) are the main targets of CAR-T engineered therapy [24]. A critical therapeutic barrier is the diverse expression of TAAs in GBM cells. In addition to HER2, expression levels of other antigens in GBM impair the function of HER2-CAR-T because the diversity of GBM cell antigens makes it impossible to identify HER2 antigens, thereby affecting the efficiency of tumor treatment. To date, a series of CAR-T therapeutic targets have been identified toward GBM, including epidermal growth factor receptor variant III, interleukin-13 receptor subunit alpha-2, HER2, B7 homolog 3 protein, CD70, disialoganglioside, matrix metalloproteinase-2, and natural killer group 2, member D [14]. Exploring the targeting of multiple TAAs on GBM by identified CAR-T cells, including the co-expression of several CARs on a single T cell and the expression of a chimeric receptor including two or more antigen recognition domains, which in turn leads to the identification of multiple antigens through individual receptors [25], are both more effective treatments for GMB to overcome tumor heterogeneity. Combining CAR-T treatment with other immunotherapies is a meaningful strategy for tumor therapy. In hematological malignancies, a combination of programmed cell death protein 1 blockade and CD19 CAR-T cell therapy in patients with B-ALL improves outcomes and CAR-T cell persistence [26]. A recent study has shown that third-generation HER2-specific CAR-T cells can efficiently eliminate GBM cells in vitro and that the activity of the administered CAR-T cells is increased by their combination with programmed cell death protein 1 blockade [27].

Before CAR-T cells can function, they must bypass the blood-brain barrier, a non-fenestrated physical barrier comprising specialized capillary endothelial cells interconnected by multi-protein tight junctions [28]. Unlike hematological malignancies, the peripheral blood is not a compartment of therapeutic action, and the effective CAR-T cell dose and frequency/schedule of administration for GBM are elusive. In addition, a major concern when targeting HER2 is the potential side effects of HER2 expression in various normal tissues, especially in vital organs, although this has rarely been an issue with HER2-specific CAR-T cell administration in humans. During CAR-T cell treatment, a high accumulation of CAR-T cells occurs in the normal lung and abdominal/mediastinal lymph nodes, on which HER2 is expressed, although at a low level [27], which could reduce the efficiency of tumor treatment. Higher infusion of many CAR-T cells can trigger the release of life-threatening supraphysiological levels of proinflammatory cytokines, which can cause serious side effects and disorders. Taken together, innovations in CAR-T cell delivery are critical for GBM therapy. We established a peritumoral injection strategy for CAR-T cells using a CDX mouse model. Compared with the traditional approach using *i.v*., we showed that peritumoral injection of CAR-T cells primed locoregional immunity more efficiently for GBM therapy. Furthermore, CAR-T therapy via peritumoral injection can be combined with novel strategies to enhance CAR-T cell cytotoxic ability to GBM.

CAR-T cells can be classified into four generations, with next- or fifth-generation CARs currently under active development [25]. Herein, engineered third-generation CAR-T cells were utilized, which combined the 4-1BB and CD28 signaling domains to provide superior activation and proliferation capacities compared with second-generation CAR-T cells. However, overstimulation of T-cell activity by two costimulatory molecules can sharply increase cytokine secretion, leading to cytokine release syndrome (CRS). Recently, the original CD3ζ has been replaced with three peptide chains of CD3, γ, δ, and ε, which can be used to solve the problem of T-cell depletion, exhaustion, and CRS during CAR-T treatment [29]. Among all CAR activations, BB-ζ secreted the highest level of cytokines, and a large portion of cytokines was related to CRS, suggesting that the use of CAR with other three peptide chains could reduce the occurrence of CRS and enhance the safety of CAR-T therapy [29]. A CAR that uses one of the signaling domains of another peptide chain rather than the ζ chain could mitigate or prevent the shortcomings of existing CAR-T cell therapies [29]. Further research should focus on the therapeutic efficacy of other peptide chains of CD3. Fourth-generation CAR-T cells incorporate cytokines or costimulatory ligands to further enhance the T-cell response or suicide genes, causing CAR-T cells to self-destroy if needed. The regulatory elements of suicide genes can increase the safety and targeting of CAR-T therapy. Fourth-generation CAR-T cells, redirected for universal cytokine killing, can secrete specific cytokines (mainly IL-12) into the tumor region, thereby modifying the tumor microenvironment and recruiting and activating other immune cells for the immune response. The fifth-generation CAR-T technology will overcome individual limitations to be universal and produced on a large scale, enabling treatment among different individuals. Therefore, strategies to achieve a more balanced immune response should be considered in the development of new CAR-T therapies.

## Data Availability

The data are available from the corresponding author on reasonable request.
